# Marine Group II Dominates Planktonic Archaea in Water Column of the Northeastern South China Sea

**DOI:** 10.3389/fmicb.2017.01098

**Published:** 2017-06-15

**Authors:** Haodong Liu, Chuanlun L. Zhang, Chunyan Yang, Songze Chen, Zhiwei Cao, Zhiwei Zhang, Jiwei Tian

**Affiliations:** ^1^State Key Laboratory of Marine Geology, Tongji UniversityShanghai, China; ^2^Department of Ocean Science and Engineering, Southern University of Science and TechnologyShenzhen, China; ^3^CNOOC Gas and Power GroupBeijing, China; ^4^School of Life Sciences and Technology, Tongji UniversityShanghai, China; ^5^Physical Oceanography Laboratory, Ocean University of ChinaQingdao, China

**Keywords:** South China Sea, Marine Group II, AOA, planktonic archaea, heterotrophic bacteria

## Abstract

Temperature, nutrients, and salinity are among the important factors constraining the distribution and abundance of microorganisms in the ocean. Marine Group II (MGII) belonging to Euryarchaeota commonly dominates the planktonic archaeal community in shallow water and Marine Group I (MGI, now is called Thaumarchaeota) in deeper water in global oceans. Results of quantitative PCR (qPCR) and 454 sequencing in our study, however, showed the dominance of MGII in planktonic archaea throughout the water column of the northeastern South China Sea (SCS) that is characterized by strong water mixing. The abundance of ammonia-oxidizing archaea (AOA) representing the main group of Thaumarchaeota in deeper water in the northeastern SCS was significantly lower than in other oceanic regions. Phylogenetic analysis showed that the top operational taxonomic units (OTUs) of the MGII occurring predominantly below 200 m depth may be unique in the northeastern SCS based on the observation that they are distantly related to known sequences (identity ranging from 90–94%). The abundance of MGII was also significantly correlated with total bacteria in the whole column, which may indicate that MGII and bacteria may have similar physiological or biochemical properties or responses to environmental variation. This study provides valuable information about the dominance of MGII over AOA in both shallow and deep water in the northeastern SCS and highlights the need for comprehensive studies integrating physical, chemical, and microbial oceanography.

## Introduction

Microorganisms are the majority of life in the ocean and play fundamental roles in ecological functions and biogeochemical cycles (Fuhrman, 2009). Advances in genomics have revealed vertical zonation of planktonic microbial communities, which reflects the nature of ocean stratification (Giovannoni et al., 1996; DeLong et al., 2006; Shi et al., 2011). For example, the photic zone is characterized by steep gradients of light, temperature (thermocline), salinity (halocline), and nutrients (neutricline), which dictate the species distribution and function in the upper water column; in the aphotic zone (>200 m depth), decreasing temperature, increasing hydrostatic pressure and lack of light and energy supplies determine the microbial community structure and function of the dark ocean (DeLong et al., 2006). However, other physical processes such as water mass movement and mesoscale eddies have also been reported to control the distribution of microbial populations or activity in the ocean (Galand et al., 2008, 2010; Zhang et al., 2009; Chen et al., 2016).

Planktonic archaea have been recognized to play important roles in global carbon and nitrogen cycles (Karner et al., 2001; Francis et al., 2005; Ingalls et al., 2006). They were initially divided into Marine Group I (MGI; now called Thaumarchaeota) and Marine Group II (MGII) that belong to Euryarchaeota (DeLong, 1992); the latter has generally been observed to dominate the surface ocean in archaeal composition, whereas the former becomes increasingly abundant at greater depths (Massana et al., 2000; Karner et al., 2001; Herndl et al., 2005; Lincoln et al., 2014). While tremendous progress has been made in the physiology, biochemistry, and ecological functions of Thaumarchaeota (Konneke et al., 2005; Ingalls et al., 2006; Martens-Habbena et al., 2009), our understanding of the MGII in the archaeal domain remains fragmented (Zhang C.L. et al., 2015). MGII have been classified into four groups (MGIIA, MGIIB, MGIIC, and MGIID) according to their 16S rRNA gene sequences (Martin-Cuadrado et al., 2015). However, there is no pure culture of MGII at the present. Recently, the metagenomic and transcriptomic studies of MGII have been increasing and gradually unveiling their potential ecological functions in carbon and nitrogen cycling in the ocean. This is exemplified by reports on the capability of MGII in degradation of protein and lipids (Iverson et al., 2012), synthesization of archaeal tetraether lipids (Lincoln et al., 2014), utilization of dissolved protein (Orsi et al., 2015), attachment and utilization of particulate organic matter (Orsi et al., 2016), and harvesting solar energy in the photic zone using proteorhodopsin (Iverson et al., 2012; Li et al., 2015; Orsi et al., 2015, 2016). However, the mechanisms controlling the distribution of MGII in different water columns of the ocean are poorly known.

The South China Sea (SCS) is the largest marginal sea of the northwestern Pacific, which has recently witnessed significant growth in microbial and biogeochemical studies in this oceanographic region (Liu et al., 2007; Moisander et al., 2008; Zhang et al., 2009; Hu et al., 2011; Wei et al., 2011; Jia et al., 2012; Jiao et al., 2014a; Tseng et al., 2015; Xia et al., 2015). The water column dynamics of the SCS is regulated by complex basin topography and water circulations resulting from East Asian Monsoon activities and the Pacific Kuroshio current intrusion (Xu and Oey, 2015; Zhang Z. et al., 2015). Occurrence of surface-confined phototrophic populations in deep waters has been observed in the western Pacific, Luzon Strait, and the SCS, which may be attributed to the active vertical mixing and isopycnal heaving of water associated with internal solitary waves, mesoscale eddies, and/or other physical processes (Jiao et al., 2014b; Chen et al., 2016). A recent study also observed the impact of asymmetrical internal solitary waves on temperature, nutrients, and chlorophyll *a* in the northern SCS (Dong et al., 2015).

Although the archaeal and bacterial community structures have been reported to be influenced by strong internal waves in the western Pacific Ocean near Luzon Strait (Jiao et al., 2014a), as well as to be affected by mesoscale cyclonic eddies in the western and the central northern SCS, the distributional patterns and niche specificity of archaea in the northeastern SCS are still unknown. The aim of this study was to unveil the relative abundance and distribution of ammonia-oxidizing archaea (AOA) and bacteria (AOB), MGII and total bacteria by targeting the archaeal and bacterial ammonia monooxygenase (*amoA*) genes and the 16S rRNA genes of MGII and bacteria, respectively, using qPCR. The archaeal community structure was also determined by using 454 sequencing. Overall, we examined that the archaeal community structure showed great similarity between different water depths in the northeastern SCS, which was different from the archaeal community structure presented in western and central regions of the SCS (Zhang et al., 2009). We also observed that the relative abundance of AOA was low throughout the whole water column (**Figures 2**, **3**) compared to other regions of the SCS (Hu et al., 2011). These results collectively suggest that the predominance of MGII in archaeal composition throughout the water column of the northeastern SCS may be caused by strong vertical mixing in this region (Tian et al., 2009; Jiao et al., 2014a).

## Materials and Methods

### Field Work and Sample Collection

**FIGURE 1:**
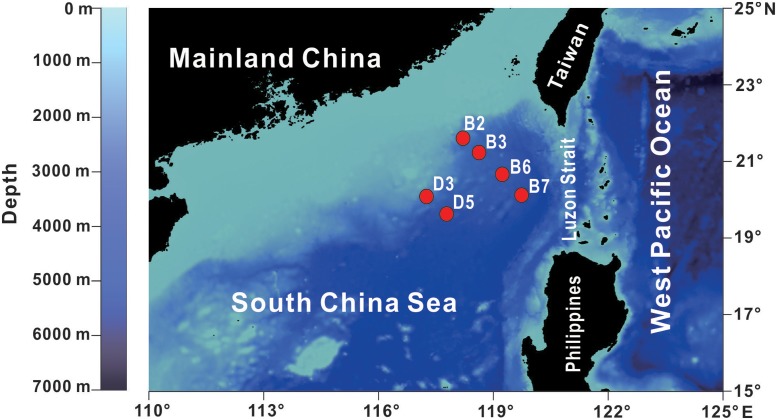
Location map for sampling stations B2 (21°51.043′N, 118°26.148′E), B3 (21°37.313′N, 118°41.565′E), B6 (20°58.589′N, 119°31.365′E), B7 (20°45.737′N, 119°48.123′E), D3 (20°02.927′N, 117°25.095′E), and D5 (19°38.843′N, 117°49.491′E) during the SCS Spring Cruise (2013). Bathymetry data was downloaded from https://www.ngdc.noaa.gov/mgg/global/. This figure was generated by Global Mapper Version 13 (http://www.bluemarblegeo.com/index.php) and CorelDRAW Graphics Suite X7 (http://www.coreldraw.com/cn/).

Twenty-four water samples were collected at different depths at the D stations (D3 and D5 with maximum depths ranging from 1800 to 3100 m, between 19°38′N and 117°49′E and 20°03′N and 117°25′E) and 49 samples at the B stations (B2, B3, B6, and B7 with maximum depths ranging from 1800 to 3200 m, between 20°45′N and 119°48′E and 21°51′N and 118°26′E) in April 2013 in the northeastern SCS (**Figure 1**). All water samples were collected by using 12-liter Niskin bottles attached to a CTD equipment; 1–2 l of sea water were filtered through a 0.22-μm membrane filter (Nitrocellulose Membrane, Millipore GSWP04700) using a vacuum pumping system, which collected both particle-associated and free-living archaea and bacteria. Filters were not exposed to air during filtration. After filtration, the membrane was preserved at -20°C immediately. Data of depth, temperature, and salinity were recorded by a CTD recorder (model SBE 9-11 Plus, SeaBird Electronics, Inc., United States).

### DNA Extraction and qPCR

Filters were cut into small pieces using sterilized scissors, which were then transferred to 2 mL tubes. DNA extraction was performed following manufacturer’s instructions provided by the FastDNA Spin Kit for Soil (MP Biomedical, Solon, OH, United States). The bacterial and MGII 16S rRNA genes and archaeal and β-AOB *amoA* genes were quantified on all samples by qPCR (PIKO REAL 96, Thermo Fisher Scientific). The abundance of each gene from each sample was normalized according to the dilution folds of DNA template and the volume of collected water. The details of primers used for qPCR were shown in **Table 1**. Each 10 μl qPCR solution consisted of 1 μl (∼1 μM) template DNA, 5 μl SYBR Premix Ex Taq^TM^ II (TaKaRa Biotechnology Co.), 0.2 μl each primer (∼1 μM), 0.1 μl Bovine Serum Albumin (BSA, 20 mg/mL) solution (TaKaRa Biotechnology Co.) and 3.5 μl deionized water. The condition was as follows: 95°C for 30 s; 40 cycles at 95°C for 5 s, 55°C for 30 s, and 72°C for 1 min. All three genes in this study were determined in triplicates for each sample. The amplification efficiency of archaeal *amoA* gene was around 95% and the R square was greater than 0.99. The amplification efficiencies of MGII and bacterial 16S rRNA genes were ∼96% and ∼85%, respectively, and the R square of them was greater than 0.99 and 0.98, respectively.

**Table 1:** Details of qPCR and sequencing primers used in this study.

Primers	Sequence (5′–3′)	Purpose	Reference
ARCH344F	ACGGGGCGCAGCAGGCGCGA	454 sequencing of archaeal 16S	Raskin et al., 1994
ARCH915R	GTGCTCCCCCGCCAATTCCT	rRNA gene	Stahl, 1991
Arch-*amoA*F	STAATGGTCTGGCTTAGACG	qPCR quantification of	Francis et al., 2005
Arch-*amoA*R	GCGGCCATCCATCTGTATGT	Crenarchaeal *amoA* gene	Francis et al., 2005
B-*amoA1*F	GGGGTTTCTACTGGTGGT	qPCR quantification of bacterial	Rotthauwe et al., 1997
B-*amoA2*R	CCCCTCKGSAAAGCCTTCTTC	*amoA* gene	Rotthauwe et al., 1997
Bac331F	TCCTACGGG AGGCAGCAGT	qPCR quantification of bacterial	Nadkarni et al., 2002
Bac797R	GGACTACCAGGGTCTAATCCTGTT	16S rRNA gene	Nadkarni et al., 2002
GII-554-f	GTCGMTTTTATTGGGCCTAA	qPCR quantification of MG II	Massana et al., 1997
Eury806-r	CACAGCGTTTACACCTAG	Euryarchaeal 16S rRNA gene	Teira et al., 2004


### 454 Pyrosequencing

Pyrosequencing of B7 and D5 samples was conducted with a Roche 454 GS FLX+ Titanium platform (Roche 454 Life Sciences, Branford, CT, United States) at the Majorbio Bio-Pharm Technology Co., Ltd. (Shanghai, China). The extracted DNA was amplified using a universal primer set ARCH344f/ARCH915r (**Table 1**). Unique barcodes for each sample were added at the 5′-end of both the forward and reverse primers to demultiplex sequences. The resulting sequences were processed using split_libraries.py-split libraries^1^ in QIIME (version 1.8.0). Sequences with quality scores greater than 20 were kept. Sequences containing ambiguous base calls or being shorter than 200 bp or with homopolymers longer than six nucleotides were discarded. Chimeric sequences were identified and removed using UCHIME (version 4.2.40^2^). Then the processed sequences were clustered using the USEARCH (UCLUST) and operational taxonomic units (OTUs) were defined at the 97% similarity cutoff by using the UPARSE (version 7.1^3^). OTU representative sequences were then selected and the taxonomy was assigned using the ribosomal database project (RDP) classifier algorithm against the SILVA (SSU115) 16S rRNA database using confidence threshold of 70% in the QIIME program. Singleton and bacterial sequences were removed. After these quality control procedures, 43,429 sequences were removed from 203,712 sequences and 160,283 high-quality sequences were produced with an average length of ∼380 bp. The OTU table was rarefied to equal sequence number for each sample basing on the least sequencing depth (*n* = 2000) sample B7_300 m (98.35% sequencing coverage) that was iterated 1000 times. The alpha diversity was calculated at the 97% identity level in QIIME, which included Shannon, Simpson, Chao1, and ace.

### Accession Numbers

The sequence data generated in this study were deposited at the Sequence Read Archive (SRA) in the National Center for Biotechnology Information (NCBI) under the BioProject accession no. SRP072671 with BioSamples SRS2071738-SRS2071762^4^.

### Methods for Data Analysis and Figure Generation

After removing singletons, 388 OTUs were obtained. The top 30 OTUs were selected to compare against blast online and their most similar reference sequences were picked out. Then these sequences were aligned by using ClustalW with default parameter settings. The Neighbor-Joining tree was constructed based on these aligned sequences with 1000 bootstrap values using the MEGA software^5^. The Bray–Curtis similarity matrix analyses were performed using the PAST^6^, with the outcome being displayed by using the HemI^7^.

## Results

Temperature and salinity profiles were similar among the B and D stations (**Supplementary Figure S1**), which are typically observed in the SCS. In this study, we focused on describing the microbiological variation in Thaumarchaeota, MGII and bacteria among these stations. The abundance of Thaumarchaeota was estimated using the archaeal *amoA* gene based on the consensus that the ratio of archaeal *amoA* gene to Thaumarchaeotal 16S rRNA gene is between 1 and 2 in the open Ocean (Church et al., 2010; Hu et al., 2011; Lund et al., 2012).

### Variation in Abundance of Thaumarchaeota, MG II, and Bacteria with Depth

**FIGURE 2:**
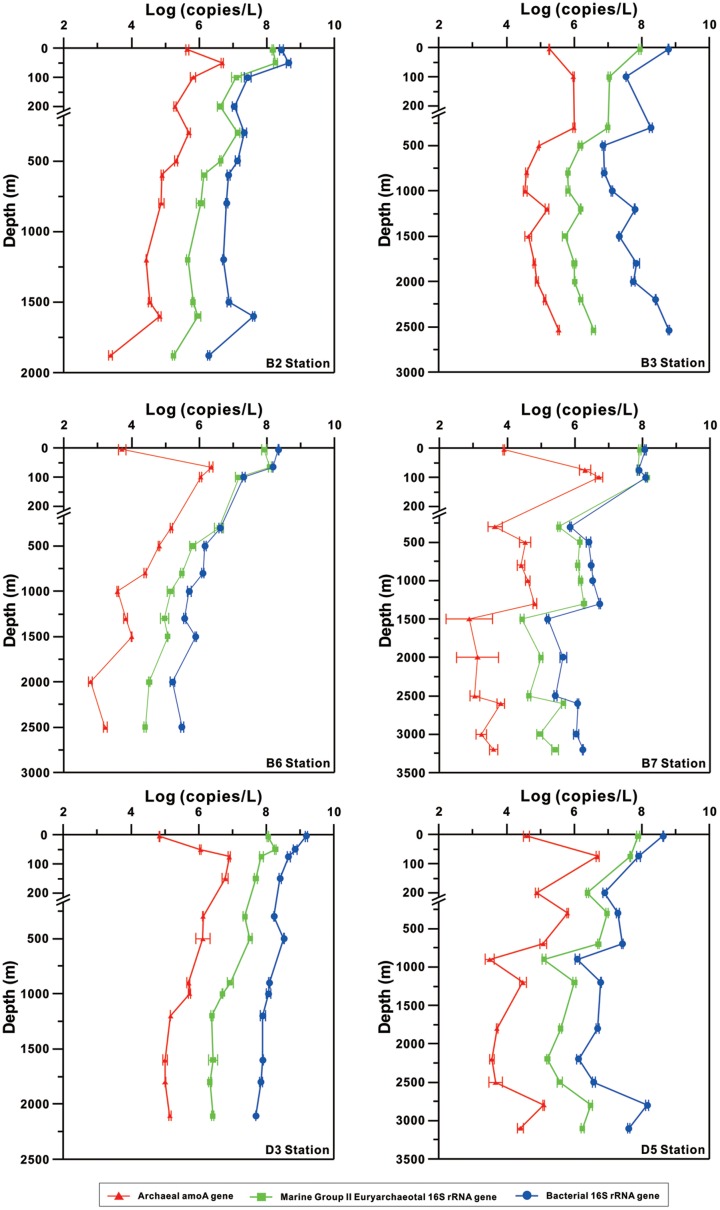
Vertical profiles of the abundances of total bacterial 16S rRNA gene, MGII 16S rRNA gene, and archaeal ammonia monooxygenase (*amoA*) gene determined by using qPCR.

The abundance of archaeal *amoA* gene was low (10^4^–10^5^ copies per liter seawater) at the surface and increased to 10^6^–10^7^ copies per liter seawater all around 100 m at the B and D stations (**Figure 2**). It then decreased to 10^3^–10^4^ copies per liter seawater below 1000–1500 m at the B and D stations; the exception was at B3, which showed consistent trend of increase again in the archaeal *amoA* gene abundance below 1500 m (**Figure 2**). The bacterial *amoA* gene depth profile was not shown because only one out of 60 samples contained detectable bacterial *amoA* gene in this study.

The MGII and bacterial 16S rRNA gene copies showed general decreasing trend with depth at all stations; exceptions were at B3 and B7, which showed increase again in abundance of both genes below about 1500 m, and at D5, which showed increase again from 2200 to 2800 m (**Figure 2**). These two genes had maximal abundance (10^8^ copies per liter seawater) at surface (5 m) or subsurface (50–100 m) and one to three orders of magnitude lower values (10^5^–10^7^ gene copies per liter seawater) at the bottom of each station; the exception again was at B7, which reached the maximal abundance of bacterial 16S rRNA gene at the bottom. The minimal copies of each gene, however, occurred at different depths at different locations.

### Distribution of Different Groups of Archaea with Depth

**FIGURE 3:**
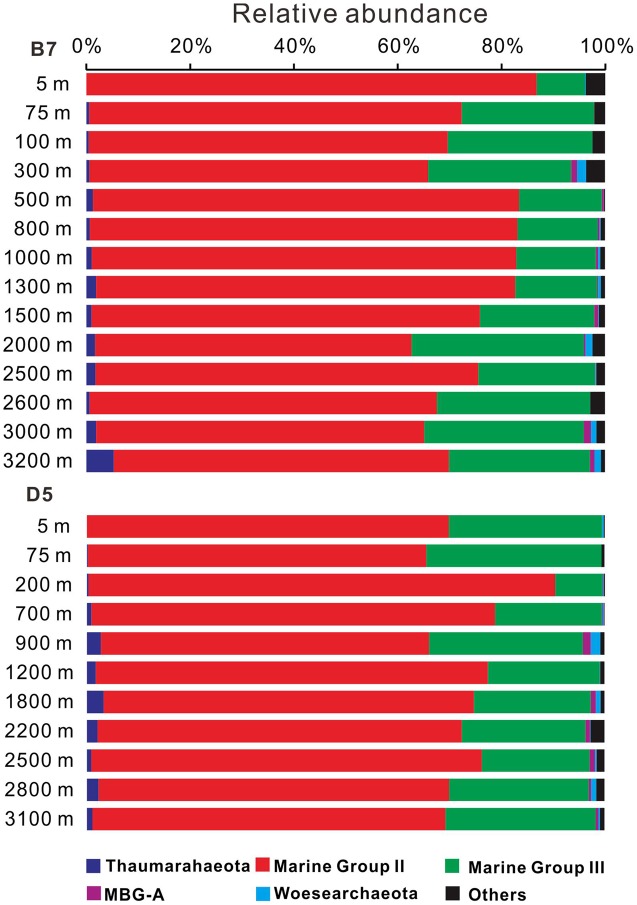
Change in archaeal 16S rRNA gene composition with depth at B7and D5 stations in the South China Sea. MBG-A represents Marine Benthic Group A. Other archaeal groups represented less than 5% of total archaeal populations.

The 16S rRNA gene sequencing analysis showed that MGII accounted for the most abundant proportion of archaea throughout the water column at both B7 and D5 stations, which were followed by MGIII; these two groups all together accounted for 91.8–99.4% of total archaeal sequences (**Figure 3** and Supplementary Table S1). The dominant clades of MGII were MGIIB and MGIIA in our study. Thaumarchaeota remained below 3.5% at all depths at these two stations, except at the 3200 m depth at B7, which exceeded 5.0% of total archaeal sequences (**Figure 3** and Supplementary Table S1).

The MBG-A (≤1.5% of total archaeal sequences), and Woesearchaeota (up to 1.8% of total archaeal sequences) were also detected at low relative abundance at the B7 and D5 stations. Other unclassified archaea collectively accounted for less than 2–4% of total archaeal sequences at these stations (Supplementary Table S1).

### Distribution of Different Groups of Archaea at the OTU Level at Different Depths

The total number of archaeal sequences was 94,495 for station B7 samples and 65,788 for D5 samples. As a result, the OTUs (at 97% cut off) were 354 for B7 samples and 339 for D5 samples. Because samples at the B7 and D5 stations showed similar vertical distribution in archaeal community composition, the sequences were combined. The top 30 archaeal OTUs from the combined B7 and D5 stations were selected for the construction of the phylogenetic tree, which represented 67% of total archaeal sequences that were similar to the percentage of the top 30 OTUs calculated separately for B7 samples (65% of total archaeal sequences) and D5 samples (70% of total archaeal sequences).

The top 30 OTUs from the combined B7 and D5 stations were all from MGII (22) or MGIII (8). The 22 MGII OTUs represented 90,826 sequences, which accounted for 78.0% of the total MGII sequences (116,391). The 8 MGIII OTUs represented 16,559 sequences that accounted for 43.8% of the total MGIII sequences (37,781).

Within the MGII, OTU-1 had the largest number of sequences (35,903) that accounted for 39.5% of total MGII sequences. OTUs-2,-3,-4, and -5 had 4.5–8.0% of total MGII sequences with a total percentage of 24.5%. The remaining OTUs had 1.2–3.5% of total MGII sequences.

**FIGURE 4:**
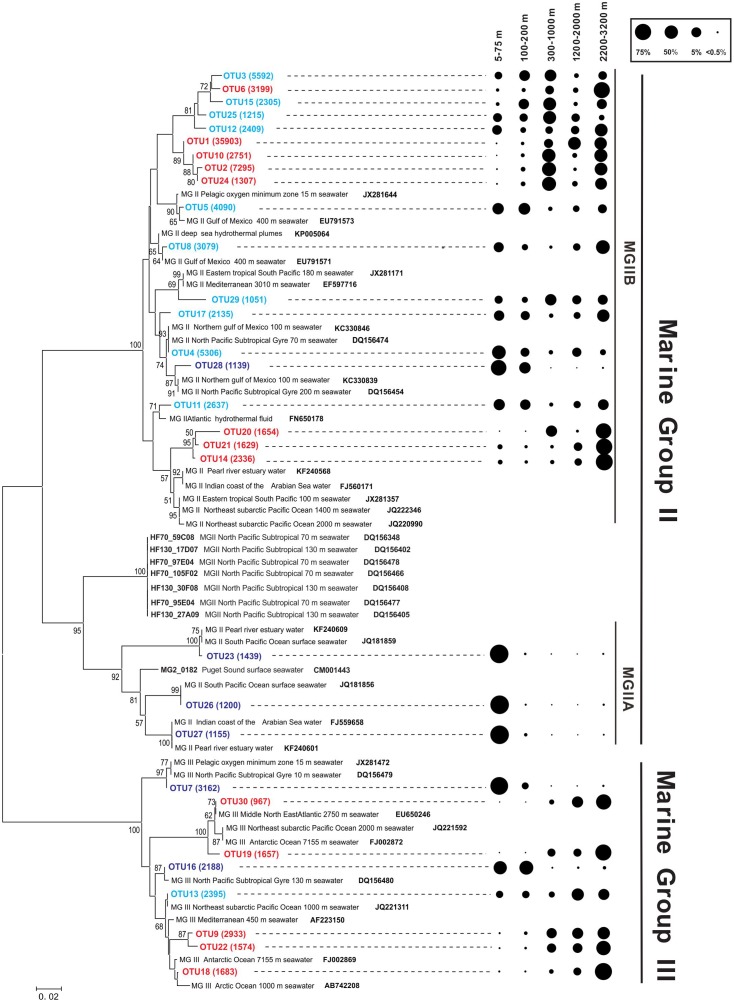
Phylogenetic tree based on the 16S rRNA gene from the Northeastern SCS (A: B7 and D5 stations). Support values, with 1000 replicates for Neighbor-Joining (NJ) analyses, were shown in the order of NJ at nodes (values lower than 50% are not shown). The numbers of environmental sequences of top OTUs recovered in this study were shown in the brackets. Dark blue OTUs indicate sequences mainly distributed in the photic zone; red OTUs indicate sequences mainly distributed below the photic zone; and cyan OTUs indicate sequences distributed relatively evenly in the photic and aphotic zones. Reference sequences from NCBI database were shown in bold. The scale bar indicates 0.02 nucleotide substitutions per site. The distribution of the dominant OTUs of MGII and MGIII is shown at right, where the circle size indicates the relative abundance of sequences in each OTU at different depth intervals.

Operational taxonomic units-1 and -2 occurred predominantly (>96% of sequences) below the photic zone (300–3200 m). OTUs-4 and -5 occurred largely (>69% of sequences) within the photic zone but also had substantial presence (18.3–20.7% of sequences) at deeper (1200–2000 m or 2200–3200 m) water depths (**Figure 4**). OTU-3 occurred more or less evenly throughout the water column. Other OTUs belonged to one of the above three categories. For example, OTUs-23, -26, -27, and -28 overwhelmingly (>99% of sequences) occurred in the photic zone, particularly the shallower (<75 m) water depths; OTUs-14, -20, and -21 occurred predominantly below 300 m, particularly in the 2200–3200 m depth interval (**Figure 4**).

Most of the MGII OTUs were closely affiliated with sequences identified from other environments (the Gulf of Mexico, the Mediterranean Sea, pelagic oxygen minimum zone, Pacific surface water, the Pearl River estuary, or the Arabian Sea). Nine of the 22 OTUs, including OTUs-1, -2, and -3, however, formed a cluster that was distantly related to sequences from other environments (**Figure 4**). These OTUs also showed a varying distribution with depth, with some of them predominantly occurring in shallow water depths, others in deeper water depths and still others occurring more or less evenly through the water column (**Figure 4**).

Within the MGIII, each OTU represented 5.8–19.1% of total sequences with an average of 12.5 ± 4.5% per OTU. Similar to the distribution of MGII OTUs, MGIII OTUs-9, -18, -19, -22, and -30 had sequences mostly occurring in the deep water, particularly in the 2200–3200 m depth interval, whereas OTUs-7 and -16 had sequences mostly occurring in the shallow water; OTU-13 had sequences occurring more or less throughout the water column, although the deeper waters tended to have greater numbers of sequences (**Figure 4**). These OTUs were similar to reference sequences from other open oceans and, unlike some of the MGII OTUs, didn’t form any unique cluster.

**FIGURE 5:**
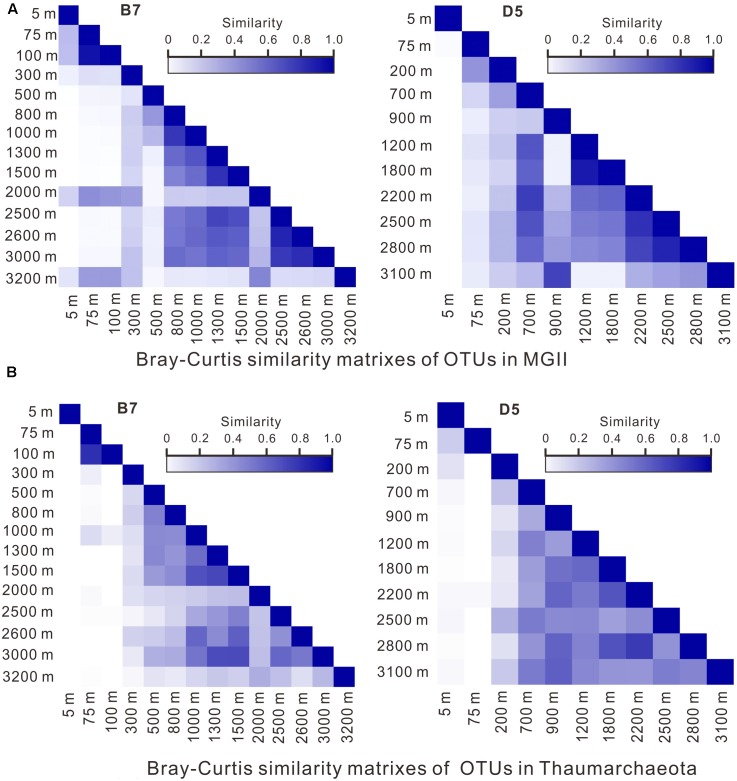
OTU similarity matrixes of MGII **(A)** and Thaumarchaeota **(B)** using the Bray–Curtis method.

Results of the Bray–Curtis analysis also demonstrated the similarity in OTUs between shallower and deeper depth intervals for both MGII and Thaumarchaeota. For example, at B7, the distribution of MGII OTUs from above 300 m showed 40–60% similarity to those from either 2000 or 3200 m depth, whereas the distribution similarity between 800 and 3000 m appeared to be much greater (50–80%). At D5, the distribution of MGII OTUs from 900 m showed about 80% similarity with the distribution of MGII OTUs from 3100 m; whereas, the distribution of MGII OTUs from 700 m showed 60–80% similarity with that from 1200 to 2800 m (**Figure 5A**). In general, the distribution similarity matrixes of OTUs in Thaumarchaeota at both stations are similar to those in MGII (**Figure 5B**), which may be attributed to similar influence by water mixing or organic matter properties, or both.

It is worth mentioning that OTUs of MGII in the top 100 m (B7) depth intervals showed greater distribution similarity than those from most depths below; OTUs of MGII from the top 5 m at D5, however, did not show distribution similarity with those from any depth below (**Figure 5A**), which suggests that surface water or water in the upper photic zone had less mixing with water from deeper depths.

We also performed the Bray–Curtis analyses on other groups (MGIII, Woesearchaea, MBG-A) of archaea, with most of the groups showing similarly well mixing features across the water column, either from surface (5 m) or below 100 m as defined above (**Supplementary Figure S2**).

### Correlation between MGII and Bacterial 16S rRNA Gene Copies

A total of 73 samples in 6 stations were included to compare the relationship between MGII and bacterial 16S rRNA gene copies. Significant correlation between the logarithmic values of the two kinds of genes existed from the surface to bottom water in the northeastern SCS (*R*^2^> 0.76, *P* < 0.01). The average logarithmic value of bacterial 16S rRNA gene copies per liter seawater was 7.23 and the average logarithmic value of MGII 16S rRNA gene copies per liter seawater was 6.39.

## Discussion

The ocean is characteristically stratified, which is reflected in much stronger difference in microbial community structure vertically than horizontally (DeLong et al., 2006; Shi et al., 2011). In this study, the distribution of Thaumarchaeota, MGII, and MGIII in the northeastern SCS (B and D stations) has distinct patterns from that in other areas of the SCS (Zhang et al., 2009; Tseng et al., 2015; Xia et al., 2015). In particular, the relatively abundant MGII and MGIII distribution with depth at the B and D stations has not been reported in previous studies that mostly describe MGII being predominant in surface water whereas Thaumarchaeota or MGIII in deep water (López-García et al., 2001; Herndl et al., 2005; DeLong et al., 2006; Galand et al., 2009; Tseng et al., 2015; Zhang C.L. et al., 2015).

Jiao et al. (2014a) have reported the presence of surface picoplankton (*Prochlorococcus*) in deep waters in Luzon Strait in the western Pacific Ocean, who dismissed aggregation, particle packing through grazing and egestion, or winter ventilation as the mechanisms for picoplankton transport to the deep water. It is also counterintuitive that organic matter is enriched in falling particles with increasing depth because the concentration of POC has been observed to decrease with depth (Dai et al., 2009). However, it is possible that the lability of organic matter differed with changing depths at these stations, as the type of organic matter can certainly influence the physiological properties of these proposed heterotrophs. More likely, the transportation of surface water microorganisms was attributed to multiple physical processes such as internal solitary waves, meso-scale eddies and turbulent mixing in the Luzon Strait and the northeastern SCS (Jiao et al., 2014a). A recent report (Chen et al., 2016) also indicated that internal solitary waves can enhance heterotrophic bacterial growth in the northern SCS. These studies highlight the importance of physical processes in controlling the distribution of planktonic microorganisms in the SCS.

The same physical processes reported by Jiao et al. (2014a) may be responsible for the transport of shallow water MGII groups down to the deep water as well as the abundant presence of MGIII throughout the water column in the northeastern SCS, which can be inferred by the similar depth profiles and similarity matrixes of different archaeal groups. Furthermore, from the available data used in the construction of the phylogenetic tree of MGII and MGIII, sequences from the SCS are more frequently affiliated with those from the Pacific Ocean than from other regions (**Figure 4**), suggesting the impact of mixing between the Pacific and the SCS waters on the community structure of planktonic archaea in the latter. On the other hand, we cannot exclude the contribution of the gravitational falling of particle-attached microbes on the occurrence of MGII at greater depths.

The low abundance of autotrophic AOA (Thaumarchaeota) throughout the whole column in the northeastern SCS is in contrast to the peak abundance of AOA occurring at 50–200 m depths, which is 5- to 10-fold higher than surface AOA observed in other regions of the SCS (Hu et al., 2011). Furthermore, the low abundance of AOA (Thaumarchaeota) compared to MGII and the scarce abundance of AOB in the northeastern SCS may be possibly due to the overall oligotrophic environment that is particularly depleted in ammonium. Hu et al. (20110) also showed the scarce AOB in SCS, in which bacterial *amoA* gene was only detected in seven out of 26 samples (Hu et al., 2011). In our study, only one out of 60 samples contained detectable bacterial *amoA* genes. AOB are commonly found in soils, freshwater, estuaries, hot springs, and marine environments (Francis et al., 2005). They appear to be more abundant than AOA in coastal settings where ammonium was relatively higher than in the oligotrophic ocean (Fan et al., 2015). AOA on the other hand are more adapted to oligotrophy in the ocean because they possess much higher affinity for ammonia than AOB (Martens-Habbena et al., 2009). It has been reported that the concentration of ammonia concentration in the area near our study sites ranged from 0.08 to 0.38 μM, which is lower than other regions of the SCS (Ling, 2011). The threshold of ammonia concentration for the growth of AOA is as low as 10 nM while the minimum ammonia concentration for the growth of AOB is greater than 1 μM observed under culture conditions (Martens-Habbena et al., 2009). Thus, the lack of ammonia probably was the main reason why AOB were almost absent in our study area. On the other hand, the low abundance of AOA throughout the whole water column in the northeastern SCS may not be due to the low abundance of ammonia alone; vertical mixing could be another reason, which could homogenize low AOA surface water with relatively more AOA abundant deeper water, a hypothesis that can be tested in future studies.

**FIGURE 6:**
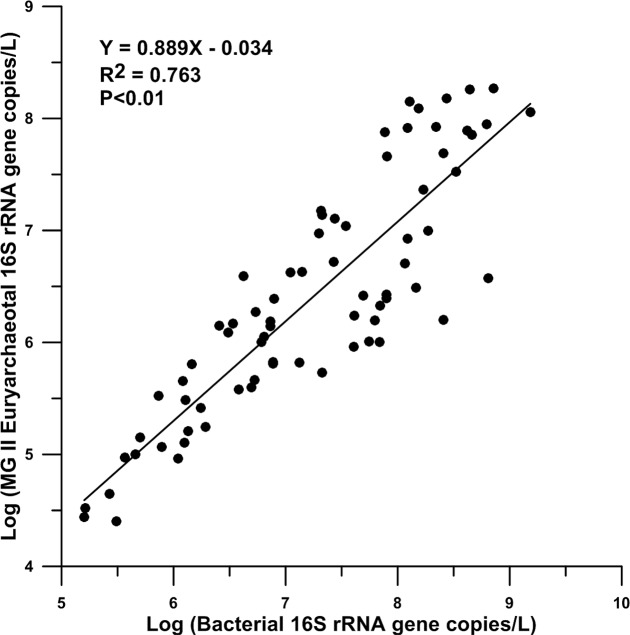
Relationship curve of the abundances of bacterial and MGII 16S rRNA genes.

It is intriguing to observe the significant correlation (*R*^2^ = 0.76, **Figure 6**) between the abundance of MGII 16S rRNA gene copies and the abundance of bacterial 16S rRNA gene copies in the northeastern SCS. Significant correlation between MGII and total bacteria has also been observed in western and northern central SCS water column (Zhang et al., 2009) and our work in the western Pacific also supports this observation (unpublished data). Thus it is likely this correlation holds true in other regions of the global ocean. At the moment, however, we can only speculate the possible mechanisms underlying this correlation. One possibility is that the occurrence of MGII and bacteria is controlled by a common variable, for example the affinity to particles. This may be supported by the observation of Orsi et al. (2015), which showed that both MGII and heterotrophic bacterial groups prefer to attach to particles in the ocean waters. Unfortunately our study only used 0.2 μm filters for sample collection, which included possibly both free-living and particle attached bacteria and MGII. Future research is needed to test this hypothesis.

In summary, our results showed that the archaeal distributional patterns in the northeastern SCS water column were distinct from other marine regions. The most abundant archaeal MGII subgroups were MGIIA (OTU 23, 26, 27 in **Figure 4**) and MGIIB (other MGII OTUs in **Figure 4**), with the former clade being mainly present at shallow waters (<100 m) while the later clade being widely present in both shallow and deep waters. In general, the heterotrophic archaea represented by MGII were much more abundant than the autotrophic archaea Thaumarchaeota throughout the whole water column in the northeastern SCS. The exact mechanisms controlling the particular archaeal distribution patterns and community structure remain unclear because of lacking direct physical and chemical measurements as well as RNA analysis. Future studies will need to couple microbiological sampling with measurements of physical and chemical properties in time series in the SCS, which should shed light on or strengthen our understanding of how heterotrophic and autotrophic archaea respond to the changing marine environment.

## Author Contributions

CZ and HL developed the idea and designed the study. HL, CY, and SC processed and analyzed the data. HL and CZ wrote the manuscript. ZZ and JT contributed to the discussion on physical processes in the SCS. ZC contributed to the data analysis.

## Conflict of Interest Statement

The authors declare that the research was conducted in the absence of any commercial or financial relationships that could be construed as a potential conflict of interest.

## Footnotes

1 http://qiime.org/scripts/split_libraries.html

2 http://drive5.com/usearch/manual/uchime_algo.html

3 http://drive5.com/uparse/

4 https://www.ncbi.nlm.nih.gov/Traces/study/?acc=SRP072671

5 http://www.megasoftware.net/

6 http://folk.uio.no/ohammer/past

7 http://hemi.biocuckoo.org/

## Supplementary Material

**FIGURE S1 |** The temperature, salinity and depth profiles of B and D stations.

**FIGURE S2 |** OTU similarity matrixes of MGIII, Woesearchaeota and MBGA using the Bray–Curtis method.
