# Supplementary figures and images for: Marine Group II Dominates Planktonic Archaea in Water Column of the Northeastern South China Sea
Source: Front Microbiol. 2017 Jun 15;8:1098. doi: 10.3389/fmicb.2017.01098 (PMC5471323; doi:10.3389/fmicb.2017.01098)

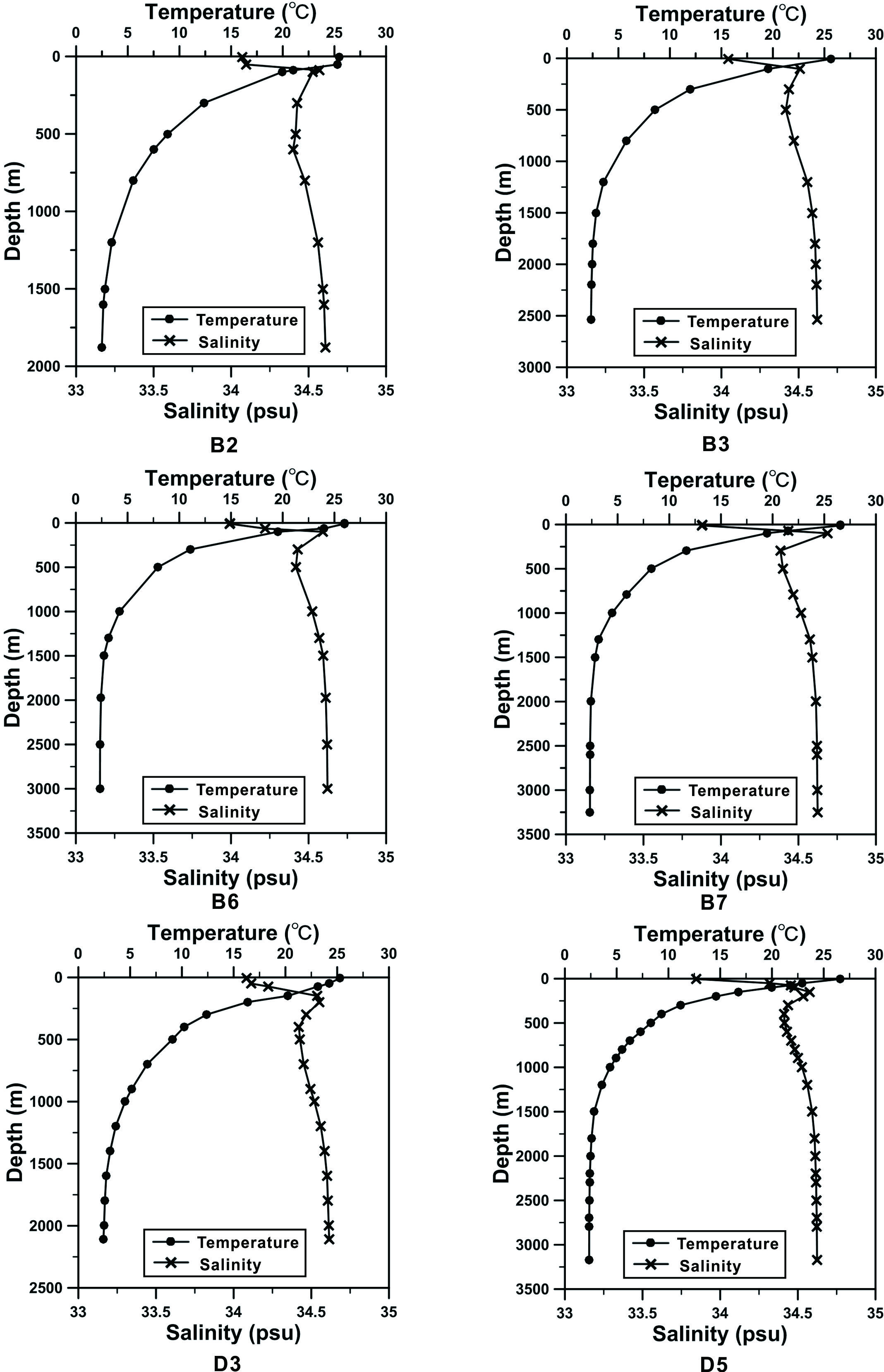

Supplement: Supplementary file 1 [file Image_1.JPEG]

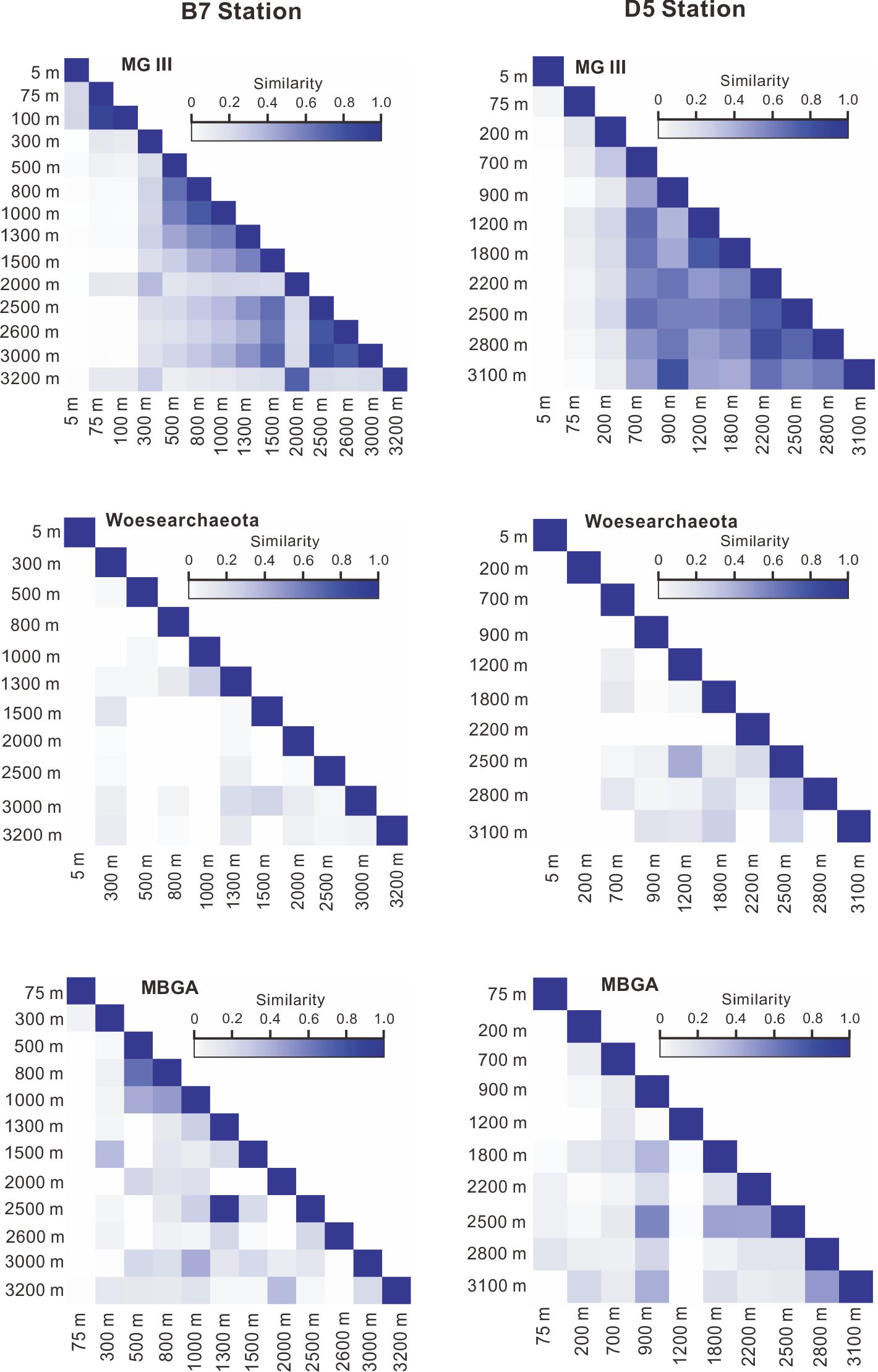

Supplement: Supplementary file 2 [file Image_2.JPEG]
